# Supplementary material for: Predicting the effect of statins on cancer risk using genetic variants from a Mendelian randomization study in the UK Biobank
Source: eLife. 2020 Oct 13;9:e57191. doi: 10.7554/eLife.57191 (PMC7553780; doi:10.7554/eLife.57191)
Supplement: Supplementary file 1. [file elife-57191-supp1.docx]

Supplementary Table 1: Definitions of site-specific cancer outcomes in UK Biobank

| **Cancer site / type** | **ICD-9 codes** | **ICD-10 codes** | **Self-report (field 20001)** | **Cancer histology (field 40011)** |
| --- | --- | --- | --- | --- |
| **Breast** | 174., 175., V10.3 | C50., Z85.3 | 1002 |  |
| **Prostate** | 185., V10.46 | C61., Z85.46 | 1044 |  |
| **Lung** | 162., V10.1 | C33., C34., C39.9, Z85.1 | 1001, 1027, 1028, 1080 |  |
| **Bowel** | 153., 154.0, 154.1, V10.05, V10.06 | C18., C19., C20., Z85.038, Z85.048 | 1020, 1022, 1023 |  |
| **Melanoma** | 172., V10.82 | C43., Z85.820 | 1059 |  |
| **Non-Hodgkin’s lymphoma** | 200., 202.0, 202.1, 202.2, 202.7, V10.71 | C82., C83., C84., C85., C86., C88.0, C88.4, Z85.72 | 1053 |  |
| **Kidney** | 189.0, V10.52 | C64., Z85.528 | 1034 |  |
| **Head/Neck** | 140., 141., 142., 143., 144., 145., 146., 147., 148., 149., 160., 161., V10.01, V10.02, V10.21, V10.22 | C00., C01., C02., C03., C04., C05., C06., C07., C08., C09., C10., C11., C12., C13., C14., C30., C31., C32., Z85.21, Z85.22, Z85.81 | 1006, 1007, 1009, 1004, 1010, 1011, 1012, 1077, 1078, 1079, 1005, 1015, 1016 |  |
| **Brain** | 191., 192.0, 192.1, 192.2, 192.3, V10.85 | C70., C71., C72.0, C72.3, Z85.841 | 1031, 1032, 1033 |  |
| **Bladder** | 188., 189.1, 189.2, V10.51, V10.53 | C67., C65., C66., Z85.51, Z85.54, Z85.53 | 1035 |  |
| **Pancreas** | 157. | C25., Z85.07 | 1034 |  |
| **Uterus** | 179., 182., V10.42, | C54., C55., Z85.42 | 1040 |  |
| **Leukaemia** | 204., 205., 206., 207., 208., V10.6 | C91., C92., C93., C94.0, C94.2, C94.3, C94.4, C94.8, C95, Z85.6 | 1048, 1055, 1056, 1074 |  |
| **Oesophagus** | 150., V10.03 | C15., Z85.01 | 1017 |  |
| **Ovaries** | 183.0, 183.2, 183.8, 183.9, V10.43 | C56., C57.0, C57.4, Z85.43 | 1039 |  |
| **Gastric** | 151., V10.04 | C16., Z85.028 | 1018 |  |
| **Liver** | 155.0 | C22.0 | 1024 | 8170, 8171, 8172, 8173, 8174, 8175 |
| **Myeloma** | 203.0, 203.1, | C90.0, C90.1 | 1050 | 9732, 9733 |
| **Thyroid** | 193., V10.87 | C73.. Z85.850 | 1065 |  |
| **Biliary** | 155.1, 156.0 | C22.1, C23., C24. | 1025 |  |
| **Cervix** | 180., V10.41 | C53., Z85.41 | 1041 |  |
| **Testes** | 186., V10.47 | C62., Z85.47 | 1045 |  |

Supplementary Table 2: Genetic variants included in gene-specific analyses for each region

| **GENE REGION** | **SNP** | **POS (HG19)** | **A1** | **A2** | **EAF** | **EXPOSURE** | | | | | **OUTCOME** | | |
| --- | --- | --- | --- | --- | --- | --- | --- | --- | --- | --- | --- | --- | --- |
|  |  |  |  |  |  | **BETA** | **SE** | | | **P** | **BETA** | **SE** | **P** |
| **HMGCR** | rs2006760 | chr5:74562029 | G | C | 0.229 | 0.053 | | 0.008 | 1.7x10^-13^ | | 0.029 | 0.007 | 8×10^-5^ |
| **HMGCR** | rs2303152 | chr5:74641707 | G | A | 0.890 | -0.042 | | 0.006 | 1.1x10^-9^ | | -0.014 | 0.010 | 0.158 |
| **HMGCR** | rs17238484 | chr5:74648496 | G | T | 0.763 | -0.063 | | 0.006 | 1.4x10^-21^ | | -0.017 | 0.007 | 0.018 |
| **HMGCR** | rs12916 | chr5:74656539 | C | T | 0.412 | 0.073 | | 0.004 | 7.8x10^-78^ | | 0.014 | 0.006 | 0.020 |
| **HMGCR** | rs10066707 | chr5:74560579 | A | G | 0.396 | 0.050 | | 0.005 | 3.0x10^-19^ | | 0.026 | 0.006 | 3×10^-5^ |
| **HMGCR** | rs5909 | chr5:74656175 | A | G | 0.100 | 0.062 | | 0.009 | 5.0x10^-13^ | | 0.022 | 0.010 | 0.030 |
| **PCSK9** | rs2479394 | chr1:55486064 | G | A | 0.281 | 0.039 | | 0.004 | 1.6x10^-19^ | | 0.001 | 0.007 | 0.892 |
| **PCSK9** | rs11206510 | chr1:55496039 | C | T | 0.172 | -0.083 | | 0.005 | 2.4x10^-53^ | | 0.002 | 0.008 | 0.764 |
| **PCSK9** | rs2149041 | chr1:55502137 | C | G | 0.823 | -0.064 | | 0.005 | 1.4x10^-35^ | | 0.005 | 0.008 | 0.474 |
| **PCSK9** | rs10888897 | chr1:55513061 | C | T | 0.680 | -0.064 | | 0.004 | 2.5x10^-50^ | | -0.002 | 0.006 | 0.784 |
| **PCSK9** | rs7552841 | chr1:55518752 | C | T | 0.596 | 0.051 | | 0.004 | 8.4x10^-31^ | | 0.008 | 0.006 | 0.214 |
| **PCSK9** | rs562556 | chr1:55524237 | A | G | 0.631 | -0.037 | | 0.004 | 5.4x10^-15^ | | -0.010 | 0.008 | 0.184 |
| **LDLR** | rs6511720 | chr19:11202306 | G | T | 0.902 | 0.221 | | 0.006 | 3.9x10^-262^ | | -0.002 | 0.009 | 0.810 |
| **LDLR** | rs688 | chr19:11227602 | C | T | 0.553 | -0.054 | | 0.004 | 1.0x10^-43^ | | -0.001 | 0.006 | 0.913 |
| **NPC1L1** | rs10234070 | chr7:44537696 | T | C | 0.899 | 0.024 | | 0.005 | 1.5x10^-6^ | | -0.010 | 0.009 | 0.280 |
| **NPC1L1** | rs2073547 | chr7:44582331 | G | A | 0.195 | 0.039 | | 0.004 | 1.9x10^-21^ | | -0.007 | 0.008 | 0.326 |
| **NPC1L1** | rs217386 | chr7:44600695 | A | G | 0.586 | -0.029 | | 0.003 | 1.2x10^-19^ | | 0.011 | 0.006 | 0.072 |
| **NPC1L1** | rs7791240 | chr7:44602589 | C | T | 0.084 | -0.034 | | 0.005 | 1.8x10^-10^ | | -0.004 | 0.010 | 0.712 |
| **NPC1L1** | rs2300414 | chr7:44682938 | A | G | 0.069 | 0.028 | | 0.006 | 5.4x10^-6^ | | -0.013 | 0.012 | 0.251 |
| **APOC3** | rs10790162 | chr11:116639104 | A | G | 0.091 | 0.231 | | 0.006 | 1.1x10^-249^ | | 0.013 | 0.012 | 0.263 |
| **APOC3** | rs603446 | chr11:116654435 | C | T | 0.553 | 0.050 | | 0.003 | 3.9x10^-43^ | | 0.012 | 0.006 | 0.038 |
| **LPL** | rs1801177 | chr8:19805708 | A | G | 0.011 | 0.164 | | 0.023 | 1.1x10^-9^ | | -0.015 | 0.023 | 0.508 |
| **LPL** | rs268 | chr8:19813529 | A | G | 0.986 | -0.280 | | 0.035 | 2.2x10^-16^ | | -0.027 | 0.022 | 0.214 |
| **LPL** | rs301 | chr8:19816934 | C | T | 0.459 | -0.109 | | 0.004 | 1.9x10^-167^ | | 0.003 | 0.007 | 0.654 |
| **LPL** | rs326 | chr8:19819439 | A | G | 0.673 | 0.087 | | 0.005 | 1.0x10^-63^ | | -0.006 | 0.006 | 0.334 |
| **LPL** | rs328 | chr8:19819724 | C | G | 0.870 | 0.167 | | 0.006 | 2.0x10^-179^ | | 0.010 | 0.010 | 0.284 |

SNP = single nucleotide polymorphism, POS (HG19) = chromosome and genomic position, A1 = effect allele, A2 = other allele, EAF = effect allele frequency, EXPOSURE = LDL-cholesterol for variants in *HMGCR*, *PCSK9* and *LDLR* gene regions, and triglycerides for *APOC3* and *LPL* gene regions, OUTCOME = overall cancer, BETA = beta-coefficient (in standard deviation units for exposure, log odds ratio for outcome) from univariable (marginal) regression analysis, SE = standard error, P = p-value.

Supplementary Table 3: Power calculations for polygenic and gene-specific analyses, representing the power to detect a given effect size (odds ratio per 1 standard deviation increase in lipid fraction) at a significance threshold of p<0.05 for overall cancer (367,703 total individuals, 75,037 cases)

| LIPID FRACTION / Gene region | ODDS RATIO = 1.05 | 1.1 | 1.15 | 1.2 |
| --- | --- | --- | --- | --- |
| Total cholesterol | 99.6% | >99.9% | >99.9% | >99.9% |
| LDL-cholesterol | 99.5% | >99.9% | >99.9% | >99.9% |
| HDL-cholesterol | 99.3% | >99.9% | >99.9% | >99.9% |
| Triglycerides | 98.3% | >99.9% | >99.9% | >99.9% |
| *HMGCR* | 11.4% | 31.4% | 58.0% | 80.6% |
| *PCSK9* | 25.7% | 72.4% | 96.3% | 99.8% |
| *LDLR* | 22.2% | 64.5% | 92.8% | 99.4% |
| *NPC1L1* | 7.2% | 16.1% | 29.5% | 45.8% |
| *APOC3* | 22.2% | 64.5% | 92.8% | 99.4% |
| *LPL* | 20.4% | 60.0% | 90.1% | 98.8% |

Supplementary Table 4: Estimates (odds ratio per 1 standard deviation increase in lipid fraction and 95% confidence interval) from polygenic multivariable Mendelian randomization analyses including all lipid-related variants. Estimates with p < 0.05 are reported in **bold**.

| CANCER SITE / CANCER TYPE | LDL-cholesterol | HDL-cholesterol | Triglycerides |
| --- | --- | --- | --- |
| Overall cancer | 1.03 (0.99-1.07) | 1.02 (0.97-1.06) | 1.02 (0.96-1.07) |
| Breast | 1.06 (0.98-1.14) | 1.06 (0.97-1.16) | 0.96 (0.87-1.07) |
| Prostate | 0.98 (0.89-1.08) | 1.03 (0.90-1.19) | 1.03 (0.90-1.19) |
| Lung | 1.05 (0.92-1.21) | 0.95 (0.81-1.12) | 0.84 (0.69-1.01) |
| Bowel | **1.16 (1.04-1.29)** | 1.08 (0.96-1.22) | 1.02 (0.87-1.18) |
| Melanoma | 1.04 (0.93-1.15) | 0.94 (0.83-1.06) | **1.18 (1.01-1.37)** |
| Non-Hodgkin’s lymphoma | 1.05 (0.91-1.23) | 1.06 (0.89-1.27) | 1.02 (0.82-1.27) |
| Kidney | 1.01 (0.83-1.23) | 0.94 (0.75-1.18) | 0.98 (0.74-1.29) |
| Head/Neck | 1.03 (0.88-1.21) | 1.03 (0.85-1.24) | 0.98 (0.78-1.24) |
| Brain | 0.98 (0.78-1.23) | 0.81 (0.62-1.07) | 0.85 (0.61-1.18) |
| Bladder | 0.99 (0.87-1.13) | 1.04 (0.89-1.22) | 1.16 (0.96-1.40) |
| Pancreas | 0.92 (0.76-1.10) | 0.89 (0.72-1.11) | 1.00 (0.77-1.30) |
| Uterus | 1.00 (0.85-1.16) | 0.96 (0.80-1.16) | 0.97 (0.78-1.21) |
| Leukaemia | 1.01 (0.85-1.20) | 1.11 (0.90-1.36) | 1.10 (0.86-1.41) |
| Oesophagus | 0.97 (0.77-1.23) | 1.08 (0.82-1.43) | 1.09 (0.78-1.53) |
| Ovaries | 1.07 (0.90-1.27) | 0.98 (0.80-1.20) | 1.09 (0.85-1.39) |
| Gastric | **0.76 (0.59-0.97)** | 1.17 (0.88-1.57) | 1.02 (0.72-1.45) |
| Liver | 0.67 (0.45-1.02) | 1.36 (0.83-2.22) | 1.12 (0.62-2.03) |
| Myeloma | 1.22 (0.93-1.59) | 1.31 (0.95-1.79) | 0.88 (0.60-1.29) |
| Thyroid | 0.89 (0.64-1.25) | 1.04 (0.70-1.55) | 0.96 (0.59-1.56) |
| Biliary | 0.86 (0.62-1.20) | 0.86 (0.58-1.28) | 0.75 (0.47-1.21) |
| Cervix | 0.97 (0.82-1.15) | 1.07 (0.88-1.30) | 1.09 (0.86-1.38) |
| Testes | 0.87 (0.68-1.11) | 0.99 (0.74-1.32) | 1.19 (0.84-1.70) |

Supplementary Table 5: Estimates from multivariable inverse-variance weighted (IVW) and MR-Egger methods for effects of lipid subfractions on overall cancer risk. In the multivariable MR-Egger method, genetic associations are orientated to the LDL-cholesterol increasing allele.

| *Overall cancer* | *Intercept* | *LDL-cholesterol* | *HDL-cholesterol* | *Triglycerides* |
| --- | --- | --- | --- | --- |
| *Multivariable IVW* | - | 1.01 (0.98-1.05) p=0.50 | 0.99 (0.95-1.03) p=0.54 | 1.00 (0.95-1.05) p=0.85 |
| *Multivariable MR-Egger* | 0.0013 (0.0008) p=0.12 | 0.99 (0.95-1.04) p=0.71 | 0.98 (0.94-1.03) p=0.44 | 0.98 (0.93-1.04) p=0.53 |

Supplementary Table 6: Multivariable Mendelian randomization estimates for HDL-cholesterol, LDL-cholesterol, and triglycerides (odds ratio with 95% confidence interval per 1 standard deviation increase in lipid fraction) from polygenic analyses for overall cancer including all lipid-associated variants excluding self-reported outcomes and restricted to the 22 named site-specific cancers

| ***Overall cancer*** | **LDL-cholesterol** | **HDL-cholesterol** | **Triglycerides** |
| --- | --- | --- | --- |
| ***All outcomes*** | 1.01 (0.98-1.05) | 0.99 (0.95-1.03) | 1.00 (0.95-1.05) |
| ***Excluding self-reported outcomes*** | 1.01 (0.98-1.05) | 0.99 (0.95-1.03) | 0.99 (0.94-1.04) |
| ***22 site-specific cancers*** | 1.03 (0.99-1.06) | 1.02 (0.97-1.06) | 1.02 (0.96-1.07) |

Supplementary Table 7: Estimates from different Mendelian randomization methods for association between bowel cancer and genetically-predicted total cholesterol and LDL-cholesterol

| *Bowel cancer* | *Multivariable IVW* | *Multivariable MR-Egger* | *Univariable IVW* | *Univariable MR-Egger* | *Weighted median* |
| --- | --- | --- | --- | --- | --- |
| *Total cholesterol* | - | - | 1.18 (1.06-1.32) | 1.10 (0.94-1.27) | 1.05 (0.91-1.22) |
| *LDL-cholesterol* | 1.16 (1.04-1.29) | 1.10 (0.97-1.26) | 1.03 (0.88-1.19) | 1.09 (0.85-1.41) | 1.04 (0.86-1.26) |

Supplementary Table 8: Number of events for each outcome and number of events that were self-reported only

| *Cancer site / type* | *Number of events* | *Number of events (excluding self reports)* | *Number (%) of events self-reported only* |
| --- | --- | --- | --- |
| *Overall* | 75,037 | 70,734 | 4303 (5.7) |
| *Breast* | 13,666 | 13,103 | 563 (4.1) |
| *Prostate* | 7872 | 7708 | 164 (2.1) |
| *Lung* | 2838 | 2774 | 64 (2.3) |
| *Bowel* | 5486 | 5220 | 266 (4.8) |
| *Melanoma* | 4869 | 3502 | 1367 (28.1) |
| *Non-Hodgkin's Lymphoma* | 2296 | 2215 | 81 (3.5) |
| *Kidney* | 1310 | 1226 | 84 (6.4) |
| *Head/neck* | 1615 | 1430 | 185 (11.5) |
| *Brain* | 810 | 655 | 155 (19.1) |
| *Bladder* | 2588 | 2461 | 127 (4.9) |
| *Pancreas* | 1264 | 784 | 480 (38.0) |
| *Uterus* | 1931 | 1611 | 320 (16.6) |
| *Leukaemia* | 1403 | 1341 | 62 (4.4) |
| *Oesophagus* | 843 | 816 | 27 (3.2) |
| *Ovaries* | 1520 | 1315 | 205 (13.5) |
| *Gastric* | 736 | 674 | 62 (8.4) |
| *Liver* | 324 | 203 | 121 (37.3) |
| *Myeloma* | 656 | 650 | 6 (0.9) |
| *Thyroid* | 375 | 114 | 261 (69.6) |
| *Biliary* | 387 | 382 | 5 (1.3) |
| *Cervix* | 1928 | 827 | 1101 (57.1) |
| *Testes* | 735 | 632 | 103 (14.0) |

Supplementary Table 9: Heterogeneity in the multivariable analyses of the lipid fractions and the univariable analyses of total cholesterol

|  | *Multivariable analysis (Lipid fractions)* | | *Univariable analysis (Total cholesterol)* | |
| --- | --- | --- | --- | --- |
| *Cancer site / type* | *Q statistic (p-value)* | *I^2^ statistic* | *Q statistic (p-value)* | *I^2^ statistic* |
| *Overall* | *296.7 (<0.001)* | *38.0* | *297.6 (<0.001)* | *38.2* |
| *Breast* | *300.3 (<0.001)* | *38.7* | *304.5 (<0.001)* | *39.6* |
| *Prostate* | *283.4 (<0.001)* | *35.1* | *284.3 (<0.001)* | *35.3* |
| *Lung* | *216.3 (0.038)* | *14.9* | *220.5 (0.030)* | *16.6* |
| *Bowel* | *250.1 (0.001)* | *26.4* | *249.7 (0.001)* | *26.3* |
| *Melanoma* | *224.9 (0.015)* | *18.2* | *236.8 (0.005)* | *22.3* |
| *Non-Hodgkin's Lymphoma* | *222.5 (0.019)* | *17.3* | *222.6 (0.024)* | *17.4* |
| *Kidney* | *207.5 (0.086)* | *11.3* | *207.9 (0.10)* | *11.5* |
| *Head/neck* | *179.5 (0.52)* | *0.0* | *179.4 (0.56)* | *0.0* |
| *Brain* | *178.2 (0.54)* | *0.0* | *179.8 (0.55)* | *0.0* |
| *Bladder* | *190.9 (0.29)* | *3.6* | *192.9 (0.29)* | *4.6* |
| *Pancreas* | *178.9 (0.53)* | *0.0* | *179.6 (0.56)* | *0.0* |
| *Uterus* | *195.1 (0.22)* | *5.7* | *195.2 (0.26)* | *5.7* |
| *Leukaemia* | *175.9 (0.59)* | *0.0* | *176.7 (0.62)* | *0.0* |
| *Oesophagus* | *193.9 (0.24)* | *5.1* | *194.3 (0.27)* | *5.3* |
| *Ovaries* | *189.0 (0.33)* | *2.6* | *190.0 (0.35)* | *3.2* |
| *Gastric* | *186.6 (0.37)* | *1.4* | *190.3 (0.34)* | *3.3* |
| *Liver* | *235.6 (0.004)* | *21.9* | *240.3 (0.003)* | *23.4* |
| *Myeloma* | *198.2 (0.18)* | *7.2* | *202.6 (0.15)* | *9.2* |
| *Thyroid* | *174.4 (0.62)* | *0.0* | *174.5 (0.66)* | *0.0* |
| *Biliary* | *176.6 (0.58)* | *0.0* | *177.4 (0.60)* | *0.0* |
| *Cervix* | *221.6 (0.021)* | *17.0* | *222.5 (0.025)* | *17.3* |
| *Testes* | *190.9 (0.29)* | *3.6* | *192.5 (0.30)* | *4.4* |

In multivariable analyses, all tests are compared to a chi-squared distribution on 181 degrees of freedom.

In univariable analyses, all tests are compared to a chi-squared distribution on 183 degrees of freedom.
